# Insulin signaling is critical for sinoatrial node maintenance and function

**DOI:** 10.1038/s12276-023-00988-0

**Published:** 2023-05-01

**Authors:** Sangmi Ock, Seong Woo Choi, Seung Hee Choi, Hyun Kang, Sung Joon Kim, Wang-Soo Lee, Jaetaek Kim

**Affiliations:** 1 Division of Endocrinology and Metabolism, Department of Internal Medicine, College of Medicine, Chung-Ang University, Seoul, Korea; 2 Departments of Physiology and Biomedical Sciences, College of Medicine, Seoul National University, Seoul, Korea; 3 Department of Physiology, Dongguk University College of Medicine, Gyeongju, Korea; 4 Division of Endocrinology and Metabolism, Departments of Internal Medicine and Biochemistry and Cell Biology, Kyungpook National University School of Medicine, Daegu, Korea; 5 Department of Anesthesiology, College of Medicine, Chung-Ang University, Seoul, Korea; 6 Division of Cardiology, Department of Internal Medicine, College of Medicine, Chung-Ang University, Seoul, Korea

**Keywords:** Heart failure, Translational research

## Abstract

Insulin and insulin-like growth factor 1 (IGF-1) signaling regulate cellular growth and glucose metabolism in the myocardium. However, their physiological role in the cells of the cardiac conduction system has never been explored. Therefore, we sought to determine the spatiotemporal function of insulin/IGF-1 receptors in the sinoatrial node (SAN). We generated cardiac conduction cell-specific inducible IGF-1 receptor (IGF-1R) knockout (KO) (CSIGF1RKO), insulin receptor (IR) KO (CSIRKO), and IR/IGF-1R double-KO (CSDIRKO) mice and evaluated their phenotypes. Telemetric electrocardiography revealed regular sinus rhythm in CSIGF1RKO mice, indicating that IGF-1R is dispensable for normal pacemaking. In contrast, CSIRKO and CSDIRKO mice exhibited profound sinus bradycardia. CSDIRKO mice showed typical sinus node dysfunction characterized by junctional rhythm and sinus pauses on electrocardiography. Interestingly, the lack of an insulin receptor in the SAN cells of CSIRKO and CSDIRKO mice caused sinus nodal fibrosis. Mechanistically, hyperpolarization-activated cyclic nucleotide-gated channel 4 (HCN4) protein expression significantly decreased in the CSIRKO and CSDIRKO mice relative to the controls. A patch-clamp study of the SAN cells of CSIRKO mice revealed a significant decrease in the funny current, which is responsible for spontaneous diastolic depolarization in the SAN. This result suggested that insulin receptor loss reduces the heart rate via downregulation of the HCN4 channel. Additionally, HCN1 expression was decreased in CSDIRKO mice, explaining their sinus node dysfunction. Our results reveal a previously unrecognized role of insulin/IGF-1 signaling in sinus node structural maintenance and pacemaker function.

## Insulin signaling: A role in controlling heart rate

The hormone insulin and the related protein insulin-like growth factor-1 (IGF-1) have previously unrecognized significance in regulating heart rate through cell signaling activities affecting the pacemaker region of the heart. Researchers in South Korea led by Wang-Soo Lee and Jaetaek Kim at Chung-Ang University, Seoul, investigated the role of receptor proteins that bind to insulin and IGF-1 in the heart’s pacemaker sinoatrial node in mice. They explored the effects of genetic interventions altering the normal levels of these receptor proteins, revealing a critical role for insulin and IGF-1 in sinoatrial node function. The results could suggest new approaches to treating sinus node dysfunction (SND), also known as sick sinus syndrome. SND is associated with increased risk of fainting, atrial fibrillation and heart failure, especially in elderly or overweight people.

## Introduction

Insulin signaling acts a key regulator of glucose metabolism through the activation of the insulin receptor (IR), predominantly in the liver, skeletal muscle, and adipose tissue^1^. Insulin-like growth factor 1 (IGF-1) binds to the highly homologous tyrosine kinase receptor IGF-1 receptor (IGF-1R) and regulates cell growth and glucose homeostasis. Although insulin and IGF-1 preferentially bind to their specific receptors, both ligands can bind with lower affinity to alternate receptors that also transduce their signals^1,2^. In the heart, deletion of IR in cardiomyocytes induced small heart size and switched cardiac substrate utilization from glucose to fatty acids^3^. Conversely, excess cardiac insulin signaling exacerbated heart failure induced by pressure overload^4^. In contrast, deletion of IGF-1R in cardiomyocytes resulted in normal postnatal cardiac size but diminished exercise-induced cardiac hypertrophy with delayed cardiac senescence phenotypes^5,6^. Strikingly, disruption of IR and IGF-1R signaling induced neonatal cardiac autophagic death^7^.

Sinus node dysfunction (SND) is a cardiac conduction disturbance characterized by profound sinus bradycardia, pauses, arrest, and sinoatrial node exit block^8^. Furthermore, SND is associated with increased syncope, heart failure, and atrial fibrillation risks. Clinically, symptomatic SND is referred to as sick sinus syndrome. In a prospective cohort study, aging and several cardiovascular risk factors, such as a high body mass index, slow heart rate, and high prevalence of hypertension, were significantly associated with sick sinus syndrome. In contrast, diabetes was only marginally related to the development of sick sinus syndrome^9^. Furthermore, individuals who developed sick sinus syndrome faced an increased risk of death and cardiovascular disease in two large community-based cohorts^10^. Early studies demonstrated that hyperglycemia per se induced SND. In a streptozotocin-induced mouse model of diabetes, the heart rate was significantly reduced, and the sinus node recovery time corrected for the heart rate was prolonged through sinus nodal pacemaker cell apoptosis^11^. Diabetic mice rescued by insulin administration showed an increased heart rate, suggesting that the absence of insulin signaling may contribute to the mechanism of SND. In addition, SND characterized by prolonged sinus node recovery time and autonomic nerve rarefaction was present in obese db/db diabetic mice^12^.

How insulin signaling to pacemaker cells affects heart rate regulation remains undefined. To dissect the specific mechanism linking IR-dependent signaling to the sinoatrial node (SAN) conduction system without the systemic effect of hyperglycemia, we generated cardiac conduction cell-restricted IR and/or IGF-1R knockout (KO) mice; this was based on the notion that insulin signaling in the heart is transduced through IR and IGF-1R. The aim was to reveal the role of insulin signaling in the SAN.

## Materials and methods

### Mice

Homozygous IGF-1R^fl/fl^ and IR^fl/fl^ mice^13–15^ were bred with Hcn4-CreErt2 or Hcn4- CreErt2;ROSA26^TomRed^ mice^16^ to generate conduction cell–specific KO mice, including IGF-1R^fl/fl^;Hcn4-CreErt2;ROSA26^TomRed^ (CSIGF1RKO), IR^fl/fl^;CreErt2;ROSA26^TomRed^ (CSIRKO), and IGF-1R^fl/fl^;IR^fl/fl^; CreErt2;ROSA26^TomRed^ (CSDIRKO) mice. To delete the locus of X-over P1 (loxP) site by activating CreErt2 in mice, 40 mg/kg tamoxifen was administered by intraperitoneal injection to the mice for 4 days. Non-floxed CreErt2;ROSA26^TomRed^ mice were also injected with tamoxifen and served as the wild-type (WT) group. Animals were fed standard chow and autoclaved water ad libitum and housed in temperature-controlled, pathogen-free facilities with a 12/12 h light/dark cycle. All animals were maintained on a C57BL/6J background, and male mice were used for all experiments. All animal experiments were conducted according to the guidelines approved by the institutional animal care and use committee of Chung-Ang University, Seoul, Korea.

### Telemetric ECG

An HD-X11 heart telemetry probe (Data Sciences International, Inc., New Brighton, MN, USA) was subcutaneously implanted into the abdomen (transmitter), chest (electrode), and carotid artery (catheter) of all animals 7 days before recording an ECG to assess adaptation and recovery. After the recovery period, ECG recordings were performed on each mouse for 3 days for cardiac rhythm analysis 3 weeks after the tamoxifen injections. Eight receiver channels were used for monitoring simultaneously.

### ECG

Mice were anesthetized with isoflurane (3% for induction and 1.5% for maintenance) via inhalation using a laboratory animal anesthesia machine (Vet Equipment Inc., Pleasanton, CA, USA). Following anesthesia, electrodes were inserted subcutaneously in the left and right forelimbs and left hindlimb in all animals 7 weeks after the administration of the tamoxifen injections. ECG recordings were performed using a PowerLab 4/20T with an animal BioAmp (ADInstruments, Sydney, Australia).

### RNA isolation and semiquantitative or quantitative reverse transcription polymerase chain reaction analysis

Total RNA was obtained from the right atrium (RA) and SAN tissues or cells using the RNeasy® Fibrous Tissue Mini Kit (Qiagen GmbH, Hilden, Germany) and the RiboEx™ Total RNA Solution (Geneall Biotechnology Co. Ltd., Seoul, Korea). First-strand deoxyribonucleic acid was synthesized from 20 ng total RNA with random primers using a SuperScript™ III First-Strand Synthesis System (#18080051; Thermo Fisher Scientific, Inc., Waltham, MA, USA). Polymerase chain reaction (PCR) analysis was performed using PCR Master Mix (#K0171; Thermo Fisher Scientific, Inc.) for semiquantitative reverse transcription PCR (RT‒PCR) and SsoFast™ EvaGreen® Supermix (#1725201AP; Bio-Rad Laboratories, Inc., Hercules, CA, USA) for quantitative RT‒PCR (qRT‒PCR). 18S rRNA was used as an internal control for normalization. Primers are listed in Supplementary Table 3. Postamplification melting curve analysis was performed to assess the specificity of the amplified PCR products, and relative quantities were calculated using the comparative cycle threshold method.

### Western blot analysis

Western blot analysis was performed as described previously^15^. RA and SAN tissues or cells were lysed with lysis buffer (20 mM Tris-HCl, pH 7.4; 1% Triton X-100; 1 mM EDTA; 30 mM 4-(2-hydroxyethyl)-1-piperazineethanesulfonic acid (HEPES); 50 mM Na_4_P_2_O_7_; 100 mM NaF) with Protease Inhibitor Cocktail (Roche Life Science, Basel, Switzerland) and phosphatase inhibitors. Lysates were incubated on ice for 15 min and then centrifuged at 13,000 rpm at 4 °C for 10 min. The supernatants were used for protein quantification by the bicinchoninic acid assay, and protein samples were separated by sodium dodecyl sulfate–polyacrylamide gel electrophoresis on a 10% gel. Protein samples were transferred to a nitrocellulose membrane with a 0.45 µm pore size and blocked with 5% skim milk. Primary antibodies were diluted in blocking solution (5% bovine serum albumin (BSA) in tris-buffered saline with 0.1% Tween® 20 [anti-HCN4; #sc-58622; Santa Cruz Biotechnology, Inc., Dallas, TX, USA] dilution 1:500); anti-HCN1 (#APC-056; Alomone Labs, Jerusalem, Israel [dilution 1:1000]); anti-phospho-Akt (Ser473) ([#4060; Cell Signaling Technology Inc., Danvers, MA, USA [dilution 1:1000]); anti-Akt (#9272; Cell Signaling Technology Inc. [dilution 1:1000]), and anti-glyceraldehyde 3-phosphate dehydrogenase (anti-GAPDH) (#2118; Cell Signaling Technology Inc. [dilution 1:2000]) primary antibodies were used according to the manufacturer’s protocols. The nitrocellulose membranes were then incubated with secondary antibodies in 5% skim milk for 1 h at room temperature, and protein bands were visualized using EzWestLumi ECL solution. Densitometric quantification was performed using Bio-Rad Image Lab Software (Bio-Rad Laboratories, Inc). GAPDH was used as a loading control.

### Histological analysis

Mice were sacrificed by cervical dislocation. Tissue preparation and staining were performed as described previously^15,17^. Isolated SAN tissues were fixed in 4% paraformaldehyde and sectioned at a thickness of 7 μm using a cryostat (Leica Biosystems, Wetzlar, Germany). Frozen SAN slides were used for hematoxylin and eosin staining, Sirius red staining, TUNEL, and immunostaining. Sirius red staining was performed by incubating the tissues in 0.1% Fast Green solution for 15 min at room temperature and then in 0.1% Fast Green solution and 0.1% Sirius red in saturated picric acid for 45 min at room temperature.

For immunohistochemical analysis of HCN4 and LC3II, peroxidase activity was blocked with 3% H_2_O_2_ in MeOH for 15 min at room temperature, blocked with 5% BSA for 1 h and treated with primary antibodies. Primary antibodies were anti-HCN4 (dilution 1:100) and anti-LC3II (#L7543; Merck KGaA, Darmstadt, Germany [dilution 1:200]). In addition, slides were incubated with biotinylated secondary antibodies and avidin–biotin complex reagents (#PK6100; Vector Laboratories, Inc., Newark, CA, USA) for 45 min at room temperature. Chromogenic detection was performed using a DAB Substrate Kit. For immunofluorescence staining of HCN4, IGF-1R, and IR, sections were blocked with 5% BSA for 1 h at room temperature, followed by overnight incubation with primary antibodies (anti-HCN4, dilution 1:50; anti-IGF-1 receptor [#sc-713; Santa Cruz Biotechnology, Inc.], dilution 1:50; and anti-insulin receptor [#AHR0271; Thermo Fisher Scientific Inc.], dilution 1:100) at 4 °C. After being washed in phosphate-buffered saline (PBS) with 0.05% Triton X-100 (PBST) for 10 min each, the slides were incubated with secondary antibodies (Alexa Fluor™ 647-conjugated goat anti-rat IgG, Alexa Fluor™ 488-conjugated goat anti-rabbit IgG, Alexa Fluor™ 488-conjugated goat anti-mouse IgG (Thermo Fisher Scientific, Inc.) for 1 h at room temperature. After being washed with PBS, the slides were mounted with diamidino-2-phenylindole (DAPI) solution. Images were obtained using a confocal microscope (LSM 700; Carl Zeiss AG, Jena, Germany). Apoptosis was determined using a colorimetric DeadEnd™ TUNEL Assay Kit (#G7130; Promega Corp., Madison, WI, USA) following the manufacturer’s instructions.

### Isolation of SAN pacemaker cells and immunocytochemistry

Mice were anesthetized with isoflurane to extract SAN tissues, and then pacemaker cells were isolated as previously described^18^. The SAN tissues were chopped into 4–5 pieces and incubated in a low-Ca^2+^ solution at 36 °C for 5 min. The low-Ca^2+^ solution contained 140 mM NaCl, 5 mM HEPES, 5.5 mM glucose, 5.4 mM KCl, 0.2 mM CaCl_2_, 0.5 mM MgCl_2_, 1.2 mM KH_2_PO_4_, 50 mM taurine, and 1% BSA; the pH was adjusted to 6.9 with NaOH. The SAN tissues were then incubated in 3 ml of enzyme solution containing collagenase type II (Worthington Biochemical Corp., Lakewood, NJ, USA), elastase (Worthington Biochemical Corp.), and protease type XIV (Sigma‒Aldrich®, Merck KGaA, Darmstadt, Germany) in a low-Ca^2+^ solution for 15–20 min. The tissue was then exposed to Kraft-Brule solution at room temperature and slowly dissociated using a wide-diameter glass pipette for 3–5 min. The dissociated cells were plated on laminin-coated coverslips (Supplementary Fig. 4). Subsequently, the samples were immunocytochemically stained or stored at 4 °C to record the *I*_f_. For immunocytochemical analysis, attached SAN pacemaker cells were fixed with 4% paraformaldehyde at room temperature for 10 min, permeabilized with 0.1% PBST for 10 min, blocked with 5% BSA, and incubated overnight at 4 °C with anti-HCN4 antibody (dilution 1:50). After being washed three times in PBS for 10 min each, the coverslips were treated with secondary antibodies (Alexa Fluor™ 488-conjugated goat anti-rat IgG) for 1 h at room temperature and then mounted with DAPI solution. Images were obtained using a confocal microscope.

### Patch-clamp electrophysiology

The *I*_f_ was recorded as previously described^19,20^. Whole-cell voltage-clamp recordings were carried out in isolated pacemaker cells from the SAN. The extracellular solution contained the following: 140 mM NaCl, 5.4 mM KCl, 1.2 mM KH_2_PO_4_, 5 mM HEPES, 5.5 mM glucose, 1 mM MgCl_2_, and 1.8 mM CaCl_2_. The pH was adjusted to 7.4 with NaOH. The current was elicited with a holding potential at −50 mV and a hyperpolarizing step to −110 mV for 1.0 s. The patch pipettes were filled with intracellular solution containing 5 mM NaCl, 135 mM KCl, 10 mM HEPES, 1 mM MgCl_2_, 0.1 mM CaCl_2_, 10 mM EGTA, 4 mM MgATP, and 5 mM pCr; the pH was adjusted to 7.2 with KOH. After the initial current recording, 0.5 mM BaCl_2_ was added to the extracellular solution to eliminate the Ba^2+^-sensitive K^+^ current. The current density was calculated by dividing pA by pF.

### Isolation of neonatal rat cardiomyocytes

Neonatal hearts were obtained from three-day-old Sprague Dawley rats and digested for isolation of cardiomyocytes as previously described^21^. Neonatal rat cardiomyocytes (NRCMs) were incubated in DMEM/M199 (1:1) containing 10% horse serum, 5% fetal bovine serum, 1% L-glutamine, 1% bromodeoxyuridine, and 1% penicillin/streptomycin. To confirm the relationship between insulin and HCN channel expression, NRCMs were treated with 200 nM insulin (#I9278; Merck KGaA, Darmstadt, Germany) with or without the PI3K inhibitor LY294002 (#L9908; Merck KGaA, Darmstadt, Germany) for 6–24 h and then analyzed by western blotting and qRT‒PCR.

### Echocardiography

Mice were anesthetized in an induction chamber using 3% isoflurane, and anesthesia was maintained with 1.5% isoflurane on a heating pad at 36 °C. Echocardiography was performed using the Vevo 770 System (FUJIFILM VisualSonics Inc., Toronto, ON, Canada) with a 30-MHz transducer in the 2-dimensional and motion-mode images^22^.

### Statistical analysis

Data are presented as the mean ± SEM. For intergroup comparisons, the data distribution was first evaluated for normality using the Shapiro‒Wilk test, a further Shapiro‒Wilk test after log-transformation, and a quantile‒quantile plot. The normally distributed data were compared using a *t* test or two-way analysis of variance. The nonnormally distributed data were analyzed using either an unadjusted Mann‒Whitney *U*-test or a Mann‒Whitney *U-*test with a Bonferroni-corrected *α* value. *P* < 0.05 was considered statistically significant. All analyses were conducted using the Statistical Package for the Social Sciences (SPSS®, Version 23, IBM Corp., Armonk, NY, USA).

## Results

### Genetic deletion of IGF-1R and IR in cardiac conduction cells

**Fig. 1:**
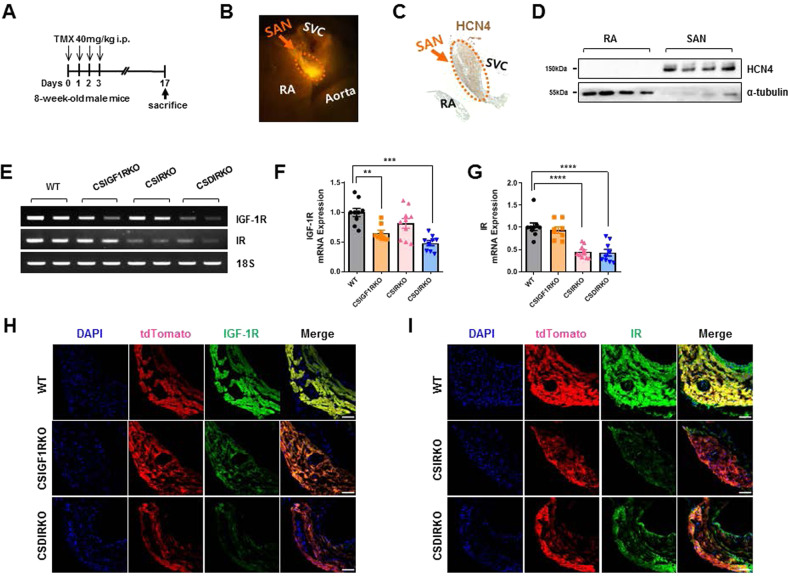
Genetic deletion of insulin-like growth factor 1 receptor (IGF-1R) and insulin receptor (IR) in cardiac conduction cells. **A** Schematic representation of tamoxifen treatment in conduction cell-specific inducible IGF-1R knockout (KO) (CSIGF1RKO), conduction cell-specific IR KO (CSIRKO), conduction cell-specific double IR/IGF-1R KO (CSDIRKO), and control mice. Eight-week-old mice were injected intraperitoneally with 40 mg/kg tamoxifen for 4 consecutive days. Two weeks after the final tamoxifen injection, the mice were sacrificed and analyzed. **B** Sinoatrial node (SAN) localization in a fluorescent image of a heart prepared from 8-week-old Hcn4-CreErt2;ROSA26^TomRed^ mice. SVC, superior vena cava; RA, right atrium. **C** Immunohistochemical staining of hyperpolarization-activated cyclic nucleotide-gated channel 4 (HCN4) in the SAN region. **D** Western blot analysis of HCN4 in the RA and SAN from Hcn4-CreErt2;ROSA26^TomRed^ mice; α-tubulin was used as a loading control. **E** Representative semiquantitative RT‒PCR analysis of *Igf-1r* and *ir* mRNA expression in the SAN of wild-type (WT), CSIGF1RKO, CSIRKO, and CSDIRKO mice. **F**, **G** Relative levels of mRNA expression were normalized to the levels of 18 S rRNA; the expression level in WT mouse hearts was arbitrarily set to 1. Group sizes: WT (*n* = 9), CSIGF1RKO (*n* = 8), CSIRKO (*n* = 10), CSDIRKO (*n* = 9). **H**, **I** Representative immunofluorescence images of IGF-1R (green), IR (green), tandem dimer Tomato (tdTomato) (red), and DAPI (blue) signal in the SAN of WT, CSIGF1RKO, CSIRKO, and CSDIRKO mice. Magnification ×20; scale bar, 50 μm. Data are presented as the mean ± SEM. **P < 0.01; ***P < 0.001; ****P < 0.0001.

To elucidate the role of insulin signaling in SAN pacemaker cells, IGF-1R^fl/fl^, IR^fl/fl^, or IGF-1R^fl/fl^; IR^fl/fl^ mice^13–15^ were bred with hyperpolarization-activated cyclic nucleotide-gated channel 4-cyclization (Cre) recombinase-estrogen receptor T2 (Hcn4-CreErt2)^23^ or Hcn4-CreErt2;reverse orientation splice acceptor 26 (ROSA26^TomRed^) mice^16^ to generate IGF-1R^fl/fl^;Hcn4-CreErt2;ROSA26^TomRed^ (conduction cell-specific IGF-1 receptor [IGF-1R] knockout [KO] [CSIGF1RKO]), IR^fl/fl^;Hcn4-CreErt2;ROSA26^TomRed^ (conduction cell-specific IR KO [CSIRKO]), and IGF-1R^fl/fl^;IR^fl/fl^;Hcn4-CreErt2;ROSA26^TomRed^ (conduction cell-specific double IR/IGF-1R KO [CSDIRKO]) mice. ROSA26 reporter tandem dimer Tomato (tdTomato) mice produce uniform red fluorescence when exposed to Cre recombinase. We injected tamoxifen for 4 consecutive days to activate Hcn4-CreErt2 and sacrificed the mice 14 days after the tamoxifen injections (Fig. 1A).

Figure 1B shows the anatomic boundaries of the mouse SAN. We dissected the HCN4-expressing fluorescent SAN and the surrounding nonfluorescent right atrial tissues under a fluorescence microscope. The hyperpolarization-activated cyclic nucleotide-gated channels (HCNs) comprise a depolarizing funny current (*I*_f_) that contributes to SAN pacemaking^24^. Although HCN isoforms HCN1–4 are present in human SAN cells, mouse SAN tissues express HCN1 and HCN4 proteins exclusively. However, neither HCN1 nor HCN4 protein was found in the right atrium^25,26^. Therefore, we confirmed that tdTomato successfully marked SAN tissue, as verified by immunohistochemistry (Fig. 1C) and western blotting (Fig. 1D). Messenger ribonucleic acid (mRNA) expression of IGF-1R in the SAN was reduced to 39% (*P* < 0.01) and 67% (*P* < 0.001) of the level of WT in CSIGF1RKO and CSDIRKO mice, respectively (Fig. 1E, F). In addition, IR mRNA levels were reduced to ~65% in CSIRKO and CSDIRKO mice (*P* < 0.0001) (Fig. 1E, G). The residual mRNA expression was likely from non-pacemaker cells within SAN tissue, such as adipocytes and fibroblasts.

We further investigated whole-mount immunostained SAN tissues and confirmed diminished expression of IGF-1R in CSIGF1RKO and CSDIRKO and decreased IR expression in the SAN of CSIRKO and CSDIRKO mice (Fig. 1H, I). Surprisingly, the abundance of the tdTomato reporter was attenuated in the SAN of CSIRKO and CSDIRKO but not CSIGF1RKO mice, suggesting that insulin signaling, but not IGF-1 signaling, may affect HCN4 expression (Fig. 1H, I and Supplementary Fig. 1).

### Deletion of IR induces bradycardia, and the combined deletion of IGF-1R and IR causes sinus node dysfunction

**Fig. 2:**
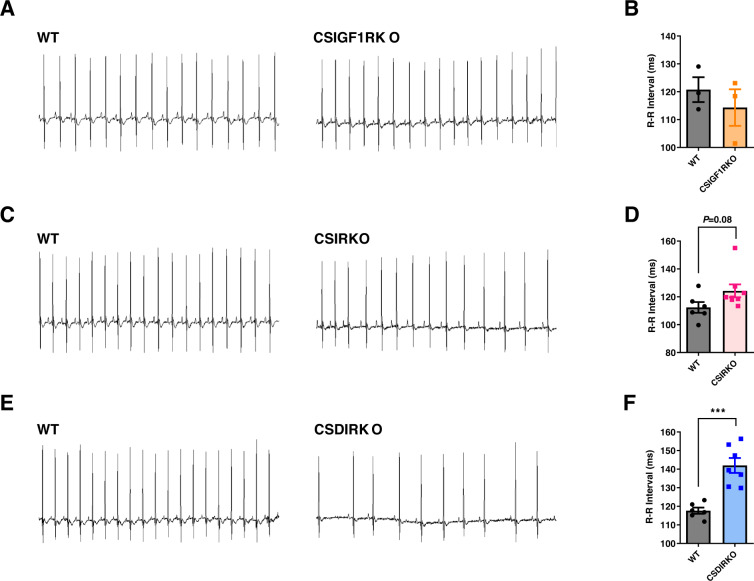
Deletion of the insulin-like growth factor and insulin receptors induces sinus bradycardia. Representative telemetric recordings of the electrocardiogram (ECG) (**A**) Quantitation of interbeat (R-R) intervals; **B** wild-type (WT) and conduction cell-specific inducible insulin-like growth factor 1 receptor (IGF-1R) knockout (KO) CSIGF1RKO mice, *n* = 3 per group. **C** Representative telemetric ECG recording; **D** quantitation of R-R intervals; WT and conduction cell-specific insulin receptor (IR) KO (CSIRKO) mice, group sizes: WT (*n* = 6) and CSIRKO (*n* = 8); **E** representative telemetric ECG recording of conduction cell-specific double IR/IGF-1R KO (CSDIRKO) mice showing sinus arrhythmia; **F** quantification of R-R intervals of CSDIRKO compared to WT mice, group sizes: WT (*n* = 6) and CSDIRKO (*n* = 7). Data are presented as the mean ± SEM. ***P < 0.001.

To determine whether impaired insulin signaling induces SND, we recorded electrocardiograms (ECGs) from freely moving mice by telemetry 3 weeks after the tamoxifen injections. ECG analysis of the CSIGF1RKO mice showed normal sinus rhythm (Fig. 2A, B). However, compared with those of the WT mice, the ECG of CSIRKO mice displayed slight interbeat (R-R) interval prolongation (Fig. 2C, D). Furthermore, a long R-R interval was statistically significant in CSDIRKO mice compared to WT mice (Fig. 2E, F).

**Fig. 3:**
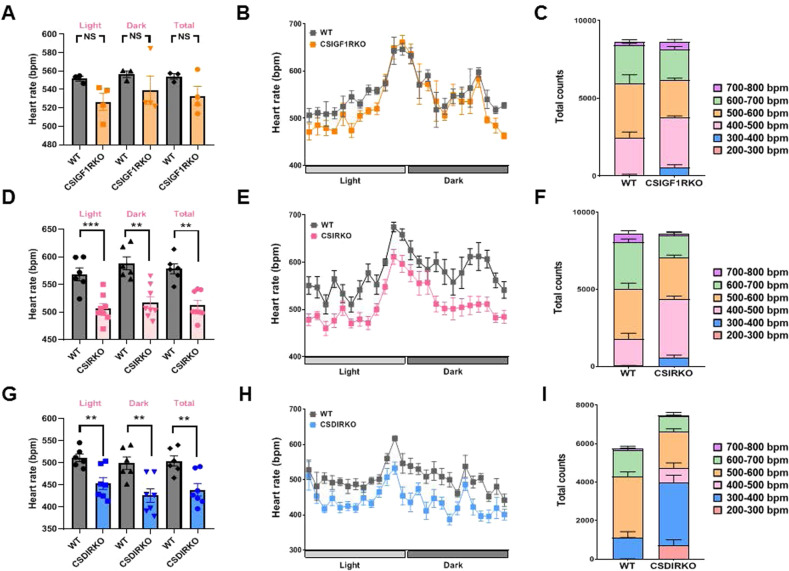
Telemetric findings and analysis of heart rate. **A**, **B** Mean heart rate with a 12 h/12 h light/dark cycle (8:00 AM to 8:00 PM) in wild-type (WT) and conduction cell-specific inducible insulin-like growth factor 1 receptor (IGF-1R) knockout (KO) (CSIGF1RKO); **D**, **E** conduction cell-specific insulin receptor (IR) KO (CSIRKO); **G**, **H** conduction cell–specific double IR/IGF-1R KO (CSDIRKO); electrocardiogram (ECG) recording over 72 h; heart rate values were analyzed at 10-s intervals. **C** Mean heart rate histograms of the WT and CSIGF1RKO mice: **F** CSIRKO; **I** CSDIRKO mice. Group sizes: WT (*n* = 3), CSIGF1RKO (*n* = 4); WT (*n* = 6), CSIRKO (*n* = 8); WT (*n* = 6), CSDIRKO (*n* = 7). Data are presented as the mean ± SEM. **P < 0.01; ***P < 0.001.

Next, we recorded heart rates for 3 days by telemetry. Baseline heart rates were similar during the light and dark phases in WT (light, 551 ± 2.6 bpm; dark, 556 ± 3.6 bpm; total 554 ± 6.1 bpm) and CSIGF1RKO (light, 526 ± 9.2 bpm; dark, 539 ± 15.1 bpm; total, 532 ± 10.3 bpm) mice (Fig. 3A, B). When the heart rates were sorted into 200 bpm intervals, histograms showed that the most common bpm range, accounting for >60% of WT and CSGF1RKO mice, was 400–600 bpm (WT, 68.3%; CSIGF1RKO, 65.6%). Moreover, 600–800 bpm accounted for 31% of WT and 28% of CSGF1RKO mice, with the least common interval being 200–400 bpm (WT, 0.7%; CSIGF1RKO, 6.0%) (Fig. 3C and Supplementary Table 1). However, CSIRKO and CSDIRKO mice displayed lower heart rates than WT mice at baseline. In particular, the mean heart rate of CSDIRKO mice was decreased compared to that of CSIRKO mice in both the light and dark phases. The heart rate of CSIRKO mice were 512 ± 8.7 bpm (light, 506 ± 8.7 bpm; dark, 517 ± 10.4 bpm) (Fig. 3D, E), while that of CSDIRKO mice was 438 ± 14 bpm (light, 452 ± 13.5 bpm; dark, 425 ± 15.4 bpm) (Fig. 3G, H). The histogram for the bpm intervals more clearly demonstrated this difference. Figure 3F, I shows that the most frequent interval in CSIRKO mice was 200–600 bpm (81.6%); for CSDIRKO mice, it was 200–400 bpm (53.5%) (Supplementary Table 1). Surprisingly, CSDIRKO, but not CSIGF1RKO or CSIRKO mice, demonstrated sinus arrest with junctional escape rhythm in the unconscious state ECG, which is characteristic of SND (Supplementary Fig. 2). Motion-mode (M-mode) echocardiography showed that CSDIRKO mice displayed preserved cardiac contractile function; this was demonstrated by fractional shortening or ejection fraction measurement (Supplementary Table 2). Therefore, CSDIRKO mice exhibit an arrhythmic phenotype with preserved cardiac contraction.

### Deletion of IR causes sinus nodal fibrosis

**Fig. 4:**
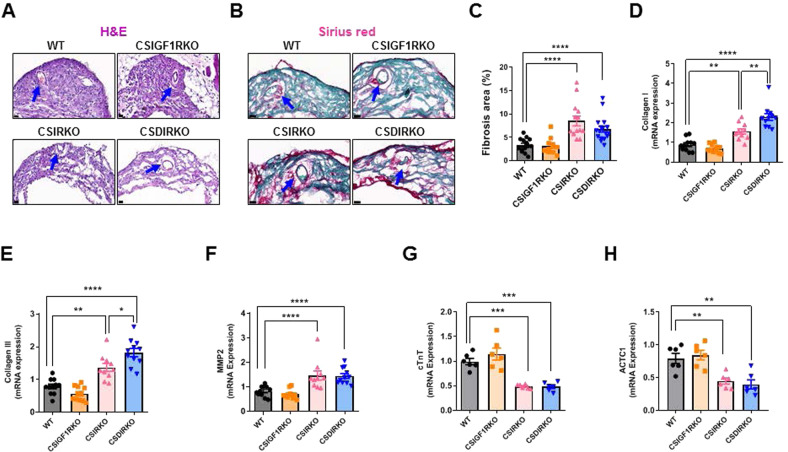
Deletion of the insulin receptor (IR) causes sinus node fibrosis. Staining of the sinoatrial node (SAN) of wild-type (WT), conduction cell–specific inducible insulin-like growth Factor 1 receptor (IGF-1R) knockout (KO) (CSIGF1RKO), conduction cell–specific insulin receptor (IR) KO (CSIRKO), and conduction cell–specific double IR/IGF-1R KO (CSDIRKO) mice at 2 weeks after four consecutive days of tamoxifen injections. **A** Hematoxylin and eosin staining (magnification ×40; scale bar, 20 μm). **B** Sirius red stain (magnification ×63; scale bar, 20 μm). Arrows indicate the sinoatrial node artery. **C** Quantification of the fibrosis area. Group sizes: WT (*n* = 11), CSIGF1RKO (*n* = 9), CSIRKO (*n* = 11), and CSDIRKO (*n* = 14). **D**–**H** mRNA quantification of *collagen I* (**D**); *collagen III* (**E**); *mmp2* (**F**); *ctnt* (**G**); and *actc1* (**H**). The results were normalized to 18S rRNA; the expression level in the WT group was arbitrarily set to 1. Group sizes: WT (*n* = 6–11), CSIGF1RKO (*n* = 6–11), CSIRKO (*n* = 6–10), CSDIRKO (*n* = 6–11). Data are presented as the mean ± SEM. **P < 0.01; ***P < 0.001; ****P < 0.0001.

Next, we examined whether the deletion of IR induces morphological changes in the SAN. As previously reported^27^, WT mouse SAN tissue comprises a compact nodal structure with densely packed nodal cells (Fig. 4A). Notably, the SAN tissue of CSIRKO and CSDIRKO mice showed a diffuse, loosely packed structure. We hypothesized that the reduced SAN density may have resulted from apoptosis or autophagy. However, there were no positive signals from terminal deoxynucleotidyl transferase-mediated dUTP nick-end labeling (TUNEL) or microtubule-associated protein 1A/1B-light chain 3-II (LC3II) immunostaining (Supplementary Fig. 3a, b). Furthermore, the mRNA expression levels of *bax*, *p62*, *vps34*, and *gabalapl1* were unchanged (Supplementary Fig. 3c–f).

We then analyzed whether fibrotic tissue replaced the myocyte composition. In CSIRKO and CSDIRKO mice, dense collagen was interspersed within the SAN (Fig. 4B, C). In addition, *collagen I*, *collagen III*, and *mmp2* mRNA levels were significantly higher in the SAN of CSIRKO and CSDIRKO mice than in that of WT mice (Fig. 4D–F). However, the myocyte marker genes *ctnt* and *actc1* showed decreased expression in the SAN tissue of CSIRKO and CSDIRKO but not CSIGF1RKO mice (Fig. 4G, H). Together, these results suggest that SAN fibrosis may contribute to the development of SND in CSIRKO and CSDIRKO mice.

### Insulin signaling regulates HCN expression

**Fig. 5:**
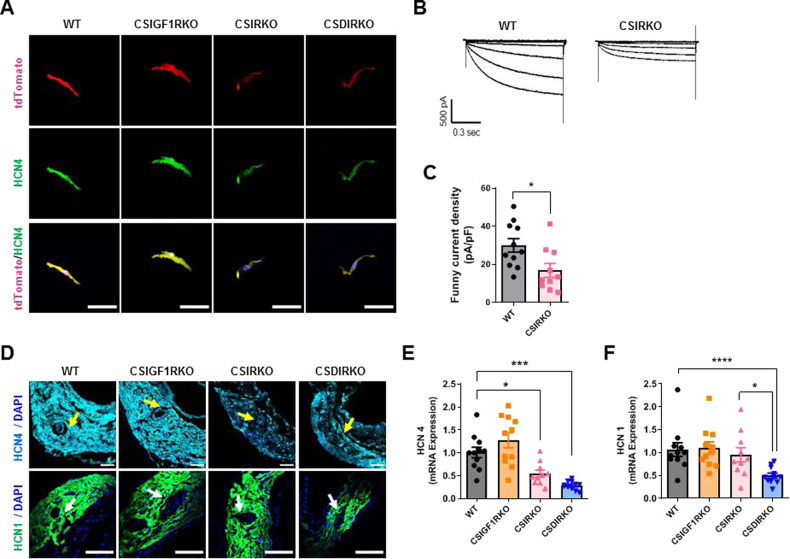
Insulin signaling regulates hyperpolarization-activated cyclic nucleotide-gated channel (HCN) expression. **A** Representative images of HCN4 immunocytochemistry sinoatrial node (SAN) pacemaker cells isolated from wild-type (WT), conduction cell–specific inducible insulin-like growth factor 1 receptor (IGF-1R) knockout (KO) (CSIGF1RKO), conduction cell–specific insulin receptor (IR) KO (CSIRKO), and conduction cell–specific double IR/IGF-1R KO (CSDIRKO) mouse SAN tissue labeled with tandem dimer Tomato (tdTomato) red fluorescence. Magnification ×40; scale bar, 50 μm. **B**, **C** The mean current density (pA/pF) from the CSIRKO mouse SAN cells (16.8 ± 3.61, *n* = 10) was significantly lower than the current density recorded from wild-type (WT) pacemaker cells (29.9 ± 3.54, *n* = 11). **D** Representative images of HCN1 and HCN4 immunostaining of WT, CSIGF1RKO, CSIRKO, and CSDIRKO mouse SAN. *Arrows* indicate the sinoatrial node artery. (HCN4, magnification ×20; scale bar, 50 μm; HCN1, magnification 20×; scale bar, 100 μm). **E**, **F** mRNA quantification of *hcn4* (**E**) and *hcn1* (**F**). The results were normalized to 18S rRNA; the expression level in the WT group was arbitrarily set to 1. Group sizes: WT (*n* = 11), CSIGF1RKO (*n* = 11), CSIRKO (*n* = 10), and CSDIRKO (*n* = 11). Data are presented as the mean ± SEM. *P < 0.05; ***P < 0.001; ****P < 0.0001.

To further explore the mechanism of profound sinus bradycardia in CSIRKO and CSDIRKO mice, we evaluated the HCN channels based on our observation that tdTomato red fluorescence was attenuated in the SAN of CSIRKO and CSDIRKO mice. As expected, tdTomato positivity colocalized with HCN4-positive cells, and the fluorescence intensity was decreased in pacemaker cells isolated from the SAN tissues of CSIRKO and CSDIRKO mice (Fig. 5A). Next, we performed patch-clamp recordings of *I*_f_ from isolated pacemaker cells. As shown in Fig. 5B, C, the amplitude of *I*_f_ was significantly reduced in CSIRKO mice compared to WT mice (mean current density, current amplitude divided by membrane capacitance [pA/pF]; 29.9 in WT and 16.8 in CSIRKO mice, *P* < 0.05). To examine whether the remaining *I*_f_ in CSIRKO mice could be explained by HCN1, SAN tissue sections were immunostained for HCN1 and HCN4. Expression of the HCN4 protein was decreased in the SAN of CSIRKO and CSDIRKO mice; HCN1 expression was reduced only in the SAN of CSDIRKO mice (Fig. 5D). In addition, the mRNA levels of HCN4 were decreased by 46% in CSIRKO mice and 72% in CSDIRKO mice. However, no difference was observed in CSIGF1RKO mice compared to WT mice (Fig. 5e). Furthermore, HCN1 mRNA expression was reduced by 53% in CSDIRKO mice compared to WT mice (Fig. 5F). Together, these results suggest that insulin signaling is critical for the regulation of *HCN* genes in SAN tissues.

**Fig. 6:**
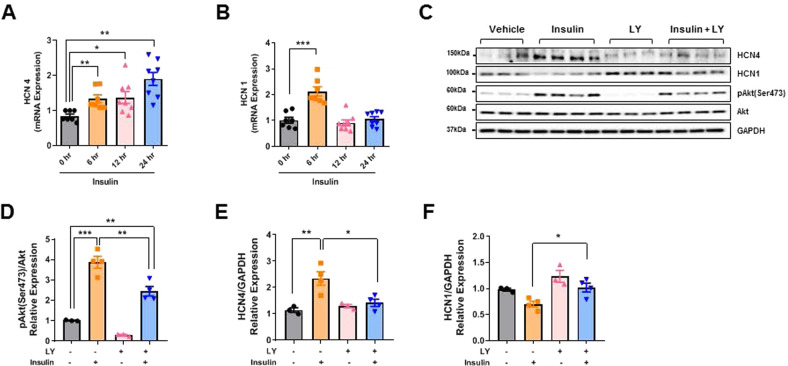
Insulin regulates hyperpolarization-activated cyclic nucleotide-gated channel (HCN) expression in cultured neonatal rat cardiomyocytes. Cells were treated with 200 nM insulin for 24 hr. mRNA quantification of *hcn4* (**A**) and *hcn1* (**B**), *n* = 7–8 per group. The results were normalized to 18S rRNA. Western blot analysis (**C**) and densitometric ratios of phosphorylated Akt at Ser473 to total Akt; (**D**), HCN4 (**E**), and HCN1 (**F**). GAPDH was used as a loading control. *n* = 3–4 per group. Data are presented as the mean ± SEM. *P < 0.05; **P < 0.01; ***P < 0.001.

To obtain direct evidence that insulin signaling regulates HCN expression, we performed an additional in vitro study using neonatal rat cardiomyocytes, which are known to express *I*_f_^28,29^. Cells were treated with 200 nM insulin for up to 24 h. Insulin upregulated HCN4 mRNA expression in a time-dependent manner. However, insulin increased HCN1 mRNA expression only at 6 h (Fig. 6A, B). Cotreatment with the phosphoinositide 3-kinase (PI3K) inhibitor LY294002 completely suppressed insulin-induced Akt phosphorylation and HCN4 protein expression (Fig. 6C–E). However, HCN1 expression was not affected by LY294002 treatment (Fig. 6F). Collectively, these data further indicated that insulin directly regulates HCN4 expression through an Akt-dependent pathway.

## Discussion

Extensive studies of the role of cardiac IR/IGF-1R using cardiomyocyte-specific Cre recombinase in mice have revealed that insulin signaling is critical for myocardial growth and substrate metabolism under physiological and pathological conditions^3,5–7,30^. However, cardiac electrical phenotypes have not been reported in mice. Although several cardiac-specific inducible Cre mouse lines target the myocardium, these lines do not specifically target the cardiac conduction system^31^. In this study, we demonstrated that conduction cell-specific KO of IR or IR/IGF-1R in adult mice leads to SAN structural remodeling through the development of nodal fibrosis. Furthermore, insulin signaling regulates sinus rhythm and rate by maintaining the *I*_f_.

The SAN is a spindle-shaped structure located in the right atrium near the opening of the superior vena cava. The SAN consists of a fibrous matrix with a densely packed population of specialized contractile cardiomyocytes. Pacemaker cells in the SAN generate an electrical impulse, propagated through an electrical conduction system, to the atrioventricular node and the ventricular myocardium for cardiac contraction. The intrinsic properties of the pacemaker cells are tightly controlled to maintain normal heart rhythm and allow adaptation to changes in physiological and pathological demands^32^. In addition, sympathetic and parasympathetic innervation to the SAN determines the heart rate. Diabetes is associated with an increased risk of arrhythmias, and sudden cardiac death is more common^33^.

Tachycardia is one manifestation of cardiac autonomic neuropathy associated with damage to the vagus nerve innervating the heart, including the SAN^34^. In db/db diabetic mice, reduced nerve density within the pacemaker tissue has been reported, as have abnormal heart rate fluctuations^12^. However, few studies have investigated sinus arrhythmia in the context of intrinsic SAN dysfunction in a diabetes model. Krishnaswamy et al. reported a baseline reduction in heart rates and SAN function in diabetic Akita mice^35^. While there is a connection between hyperglycemia and SND, a relationship between impaired insulin signaling in the SAN and normoglycemia has never been reported. Our data clearly showed that insulin signaling regulates the heart rate independently of the autonomic nervous system or hyperglycemia.

Recently, it has been reported that a streptozotocin-induced mouse model of diabetes exhibited SND, including macrophage infiltration, fibrosis, and oxidative stress, in the SAN^36^. Additionally, in the context of myocardial infarction, it has been shown that oxidized calcium/calmodulin-dependent protein kinase II mediates SAN cell apoptosis in mice with streptozotocin-induced diabetes^11^. These studies highlight the potential contribution of high-glucose-induced oxidative stress to SND. In this study, attenuated insulin signaling in the SAN did not affect apoptosis or autophagy. Notably, attenuation of insulin signaling per se resulted in SAN fibrosis. Mechanistically, it is possible that activated fibroblasts within the SAN of CSIRKO mice promoted collagen deposition. Several studies have suggested that crosstalk between pacemaker cells and fibroblasts within the SAN driven by increased expression of the secreted protein transforming growth factor (TGF)β can promote fibroblast proliferation and differentiation into myofibroblasts^37,38^. Therefore, our findings encourage further investigation into the inverse relationship between the amount of fibrosis within the SAN and heart rate.

Next, our major finding was the alteration in HCN4 expression in CSIRKO and CSDIRKO mice. Furthermore, reduced expression of HCN1 in the SAN of CSDIRKO mice indicated a more severe phenotype, such as sinus pause. Deletion of HCN4 in adult mice eliminated most of the SAN *I*_f_ current, causing cardiac arrhythmia characterized by recurrent sinus pauses and bradycardia; this suggested that HCN4 channels are essential for normal heart impulse generation and conduction^39,40^. Moreover, in a recent study, HCN1^−/−^ mice displayed congenital SND^26^. Given the SAN dysfunction following both IGF-1R and IR deletion, we speculate that both channels have compensatory effects on pacemaking activity. Although our data unveiled the critical role of insulin signaling on I_f_ current, the contribution of ion channels, such as Na^+^, Ca^2+^, and K^+^ channels, to the bradycardia phenotype in CSIRKO mice remains to be determined. Therefore, we cannot exclude the involvement of changes in other pacemaker ion channels.

In summary, this study provided compelling evidence that insulin signaling is critical for maintaining a physiological heart rate. Therefore, restoring insulin signaling is a promising therapeutic strategy to prevent or improve the treatment of SND.

### Supplementary information

Supplementary

Supplementary Fig. 4
