# Author Correction: Insulin signaling is critical for sinoatrial node maintenance and function

**DOI:** 10.1038/s12276-024-01278-z

**Published:** 2024-07-24

**Authors:** Sangmi Ock, Seong Woo Choi, Seung Hee Choi, Hyun Kang, Sung Joon Kim, Wang-Soo Lee, Jaetaek Kim

**Affiliations:** 1 Division of Endocrinology and Metabolism, Department of Internal Medicine, College of Medicine, Chung-Ang University, Seoul, Korea; 2 Departments of Physiology and Biomedical Sciences, College of Medicine, Seoul National University, Seoul, Korea; 3 Department of Physiology, Dongguk University College of Medicine, Gyeongju, Korea; 4 Division of Endocrinology and Metabolism, Departments of Internal Medicine and Biochemistry and Cell Biology, Kyungpook National University School of Medicine, Daegu, Korea; 5 Department of Anesthesiology, College of Medicine, Chung-Ang University, Seoul, Korea; 6 Division of Cardiology, Department of Internal Medicine, College of Medicine, Chung-Ang University, Seoul, Korea

**Keywords:** Heart failure, Translational research

Correction to: *Experimental & Molecular Medicine* 10.1038/s12276-023-00988-0, published online 01 May 2023

In this article the statement in the Funding information section was incorrectly given as ‘This work was supported by grants from the Basic Science Research Program through the National Research Foundation of Korea (2018R1D1A1B07046712 to S.O., 2020R1A2C1009647 to W.-S.L., and RS-2022-KH128434 (HV22C016000) to J.K.).’ and should have read ‘This work was supported by grants from the Basic Science Research Program through the National Research Foundation of Korea (2018R1D1A1B07046712 to S.O., 2020R1A2C1009647 to W.-S.L., and RS-2022-KH128434 (HV22C016000) to J.K.)’.

The original article has been corrected.

