# Supplementary material for: Insulin signaling is critical for sinoatrial node maintenance and function
Source: Exp Mol Med. 2023 May 1;55(5):965–73. doi: 10.1038/s12276-023-00988-0 (PMC10238478; doi:10.1038/s12276-023-00988-0)
Supplement: Supplementary file 1 — Supplementary [file 12276_2023_988_MOESM1_ESM.pdf]

Supplementary Fig. 1

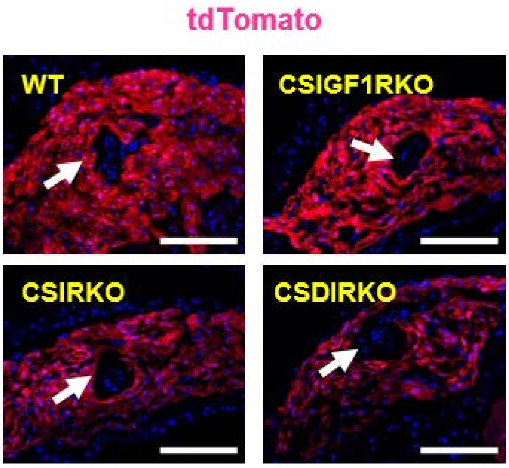

Supplementary Fig. 2

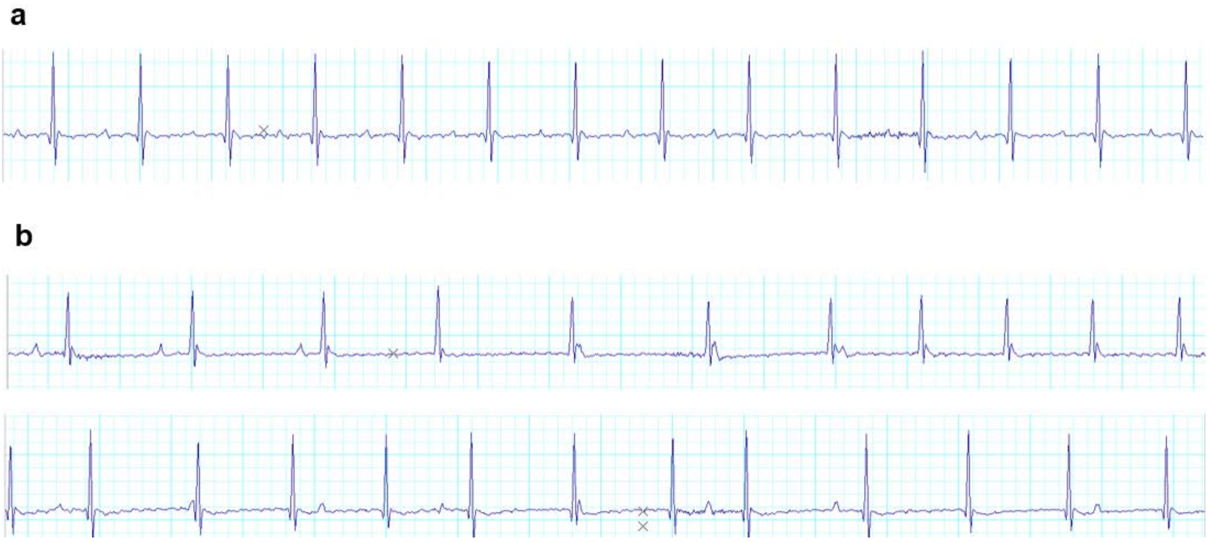

Supplementary Fig. 3

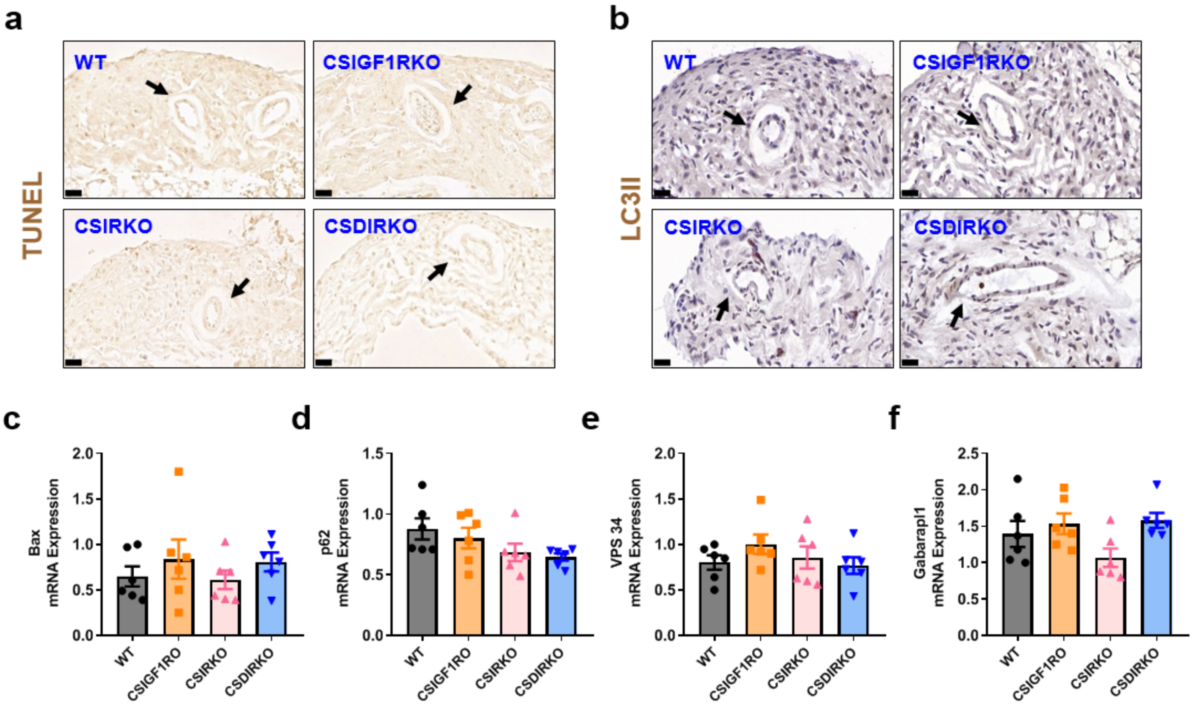

Supplementary Fig. 4

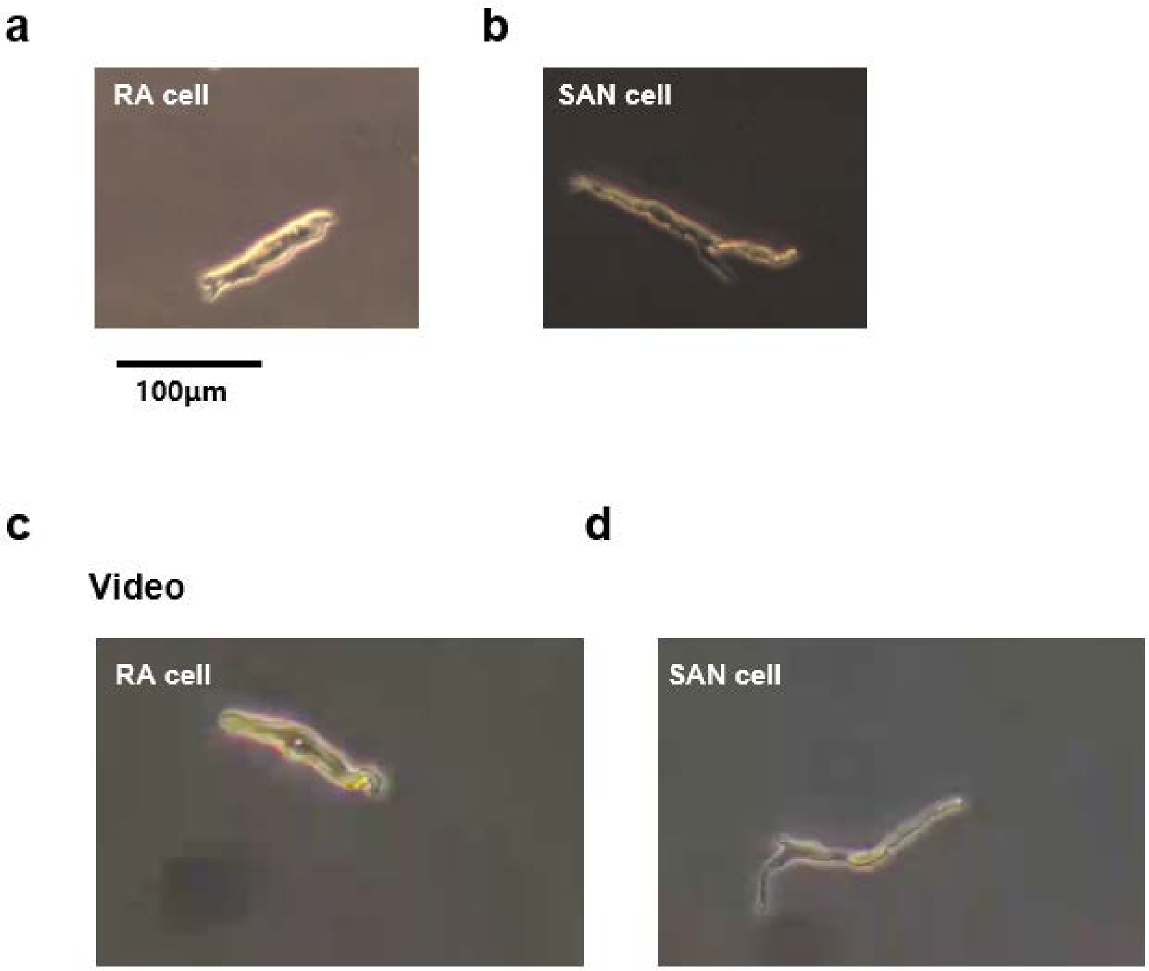

Supplementary Table 1. Mean heart rates and frequency

	Rate (bpm)			No. of mice
	Light	Dark	Total	
WT	551.7±2.6	556.6±3.6	554.2±3.1	3
CSIGF1RKO	526.5±9.2	539.3±15.1	532.9±10.3	4
WT	568.4±11.8	588.3±11.6	578.3±9.2	6
CSIRKO	506.7±8.7	517.2±10.4	512±8.7	8
WT	511.2±8.7	499±14.1	503.8±11.5	6
CSDIRKO	452.8±13.5	425.6±15.4	438.7±14	7

WT, wild type; CSIGF1RKO, conduction cell-specific inducible insulin-like growth factor 1 receptor (IGF-1R) knockout (KO); CSIRKO, conduction cell-specific insulin receptor (IR) KO; CSDIRKO, conduction cell-specific double IR/IGF-1R KO

	Frequency (%)			No. of mice
	200–400 (bpm)	400–600 (bpm)	600–800 (bpm)	
WT	0.7	68.3	31.0	3
CSIGF1RKO	6.0	65.6	28.4	4
WT	0.4	58.1	41.5	6
CSIRKO	6.6	75.0	18.4	8
WT	19.2	55.4	25.4	6
CSDIRKO	53.5	35.1	11.4	7

WT, wild type; CSIGF1RKO, conduction cell-specific inducible insulin-like growth factor 1 receptor (IGF-1R) knockout (KO); CSIRKO, conduction cell-specific insulin receptor (IR) KO; CSDIRKO, conduction cell-specific double IR/IGF-1R KO

Supplementary Table 2. Echocardiographic analysis in wild type (WT) and conduction cell-specific insulin and insulin-like growth factor 1 receptor knockout (CSDIRKO) mice 3 weeks after tamoxifen injections

	**WT (n=10)**	**CSDIRKO (n=7)**	***P* value**
LVEDD (mm)	3.89±0.08	3.87±0.15	NS
LVESD (mm)	2.87±0.11	2.91±0.13	NS
IVSd (mm)	0.81±0.02	0.81±0.03	NS
IVSs (mm)	1.18±0.01	1.18±0.03	NS
PWd (mm)	0.87±0.03	0.94±0.06	NS
PWs (mm)	1.19±0.02	1.12±0.05	NS
FS (%)	26.5±1.8	24.7±2.1	NS
EF (%)	45.6±2.6	43.1±3.2	NS

LVEDD, left ventricular end diastolic diameter; LVESD, left ventricular end systolic diameter; IVSd, interventricular septum thickness measured in diastole; IVSs, interventricular septum thickness measured in systole; PWd, left ventricular posterior wall thickness measured in diastole; PWs, left

ventricular posterior wall thickness measured in systole; FS, fractional shortening; EF, ejection fraction. Data are presented as the mean±SEM

Supplementary Table 3. Primers for semi-quantitative or quantitative reverse transcription polymerase chain reaction (RT-PCR)

(1) Semi-quantitative RT-PCR

	Gene Name	Forward Primers (5′–3′)	Reverse Primers (5′–3′)
Mice	*18s*	AAG CAT TTG CCA AGA ATG TTT T	ATG CCA GAG TCT CGT TCG TTA T
	igf-1r	CCC AGA GCA TGT ACT GTA TCC C	CAC AGC TTG GGA TTG AAA GCA A
	ir	TTG CCC AAC CAT CTG TAA GTC A	TGG GCA GAT GTC ACA GAA TCA A

(2) Quantitative RT-PCR

	Gene Name	Forward Primers (5′–3′)	Reverse Primers (5′–3′)
Mouse	*18s*	CGA AAG CAT TTG CCA AGA AT	AGT CGG CAT CGT TTA TGG TC
	hcn4	GCT GGA GGA GTA TCC CAT GA	TGT GGA GGA GGA TGG AGT TC
	hcn 1	TTGCGGTTATTACGCCTTTC	GGC GAG GTC ATA GGT CAT GT
	collagen I	ACT GGT ACA TCA GCC CGA AC	TAC TCG AAC GGG AAT CCA TC
	collagen III	ACC AAA AGG TGA TGC TGG AC	GAC CTC GTG CTC CAG TTA GC
	mmp2	TGG GGG AGA TTC TCA CTT TG	CCA TCA GCG TTC CCA TAC TT
	ctnt	CCT GCA GGA AAA GTT CAA GC	TTC CCA CGA GTT TTG GAG AC
	actc1	CGA TAT CCG CAA AGA CCT GT	AGC CAG TGC AGT GAT TTC CT
	bax	ATG GAG CTG CAG AGG ATG AT	GAA GTT GCC ATC AGC AAA CA
	p62	CCT TGC CCT ACA GCT GAG TC	CAC ACT CTC CCC CAC ATT CT
	vps34	TGG AAC TTC TGG GAA AGT GG	GGT GGG GTT GGT GTA ATG AG
	gabarapl1	TCG TGG AGA AGG CTC CTA AA	GAA CTG GCC AAC AGT GAG GT
Rat	*hcn1*	TTC ATG CAG AGG CAG TTC AC	ACC CTT TCC TGC TCC TTC TC
	hcn4	AGA TGT AGT CCA CGG GGA TG	TGG TGG AGG ACA ACA CAG AA

A, adenine; C, cytosine; G, guanine; T, thiamine

SUPPLEMENTARY FIGURE LEGENDS

Supplementary Fig. 1. Representative fluorescence images of WT, conduction cell-specific inducible insulin-like growth factor 1 receptor (IGF-1R) knockout (KO) (CSIGF1RKO), conduction cell-specific insulin receptor (IR) KO CSIRKO, and conduction cell-specific double IR/IGF-1R KO (CSDIRKO) sinoatrial node (SAN) **s**ections at 2 weeks after tamoxifen injection. *Arrows* indicate sinoatrial node artery. Magnification, 20×; scale bar, 100 μm

Supplementary Fig. 2. Electrocardiogram (ECG) of wild-type (WT) (**a**) and conduction cell-specific double insulin and insulin-like growth factor-1 receptors knockout (CSDIRKO) mice (**b**). CSDIRKO mice showed sinus arrest with junctional escape rhythm.

Supplementary Fig. 3. Apoptotic or autophagic cells were not detected in conduction cell-specific insulin receptor (IR) knock out (KO) (CSIRKO) and conduction cell-specific double IR/insulin-like growth factor-1 receptor KO (CSDIRKO) mice at 2 weeks after tamoxifen injection. Representative terminal deoxynucleotidyl transferase-mediated dUTP nick-end labeling (TUNEL) staining (**a**) and immunohistochemistry staining of microtubule-associated protein 1A/1B-light chain 3-II (LC3II) (**b**) in mouse sinoatrial node (SAN) tissue. *Arrows* indicate the sinoatrial node artery. (**c–f**) mRNA quantification of *bax* (**c**), *p62* (**d**), *vps34* (**e**), and *gabalapl1* (**f**). Results were normalized to 18S rRNA; mRNA levels were arbitrarily set as 1. n=6 per group. Magnification 63×; scale bar, 20 μm

Supplementary Fig. 4. Representative images of mouse right atrium (RA) cells (**a**) and sinoatrial node (SAN) pacemaker cells (**b**). Video recording of the beating of RA cells (**c**) and SAN pacemaker cells (**d**) from wild-type (WT) mice.
