# Supplementary figures and images for: Insulin signaling is critical for sinoatrial node maintenance and function
Source: Exp Mol Med. 2023 May 1;55(5):965–73. doi: 10.1038/s12276-023-00988-0 (PMC10238478; doi:10.1038/s12276-023-00988-0)

## Slide 1
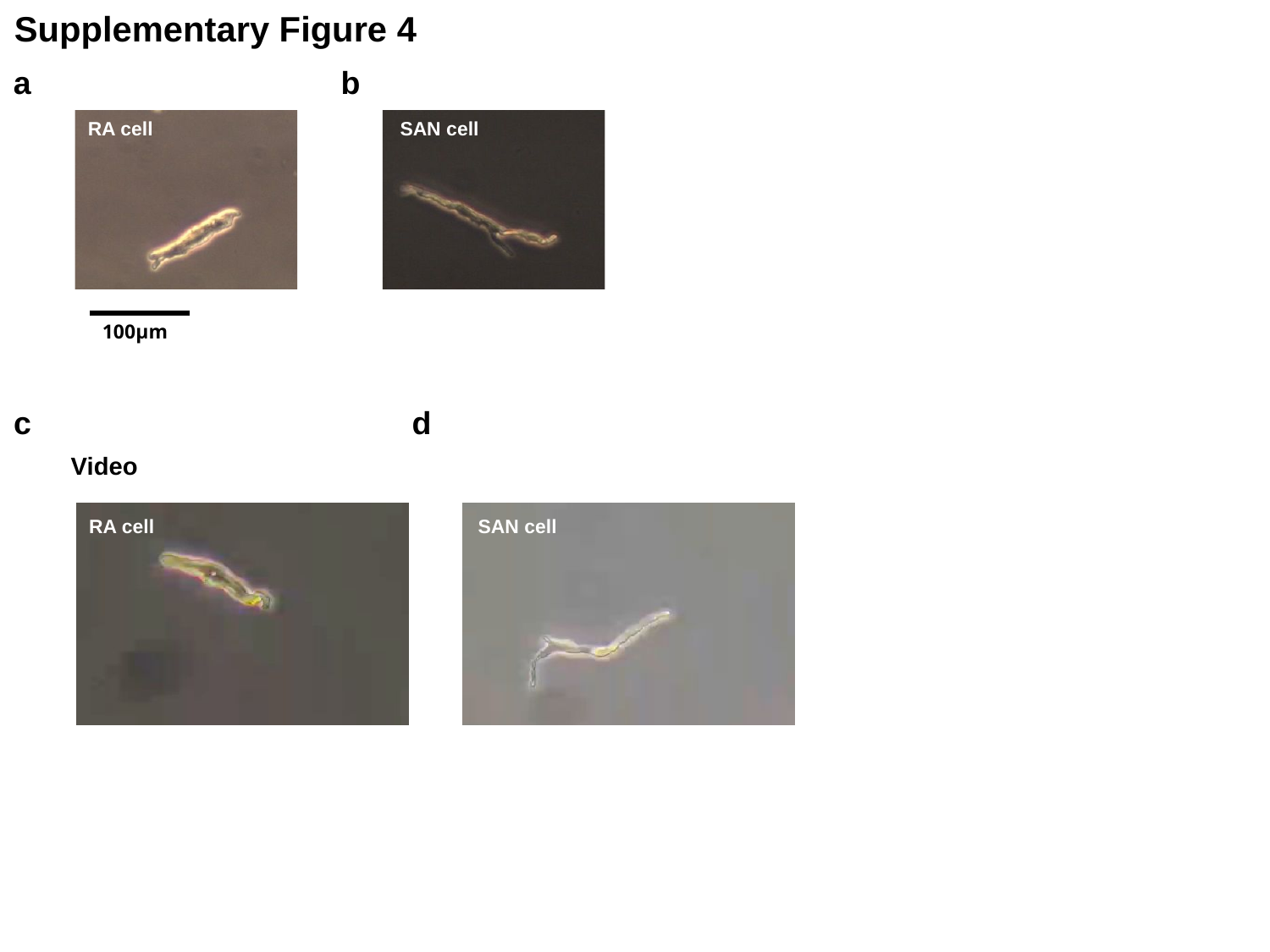

Supplementary Figure 4
a b
RA cell SAN cell
100μm
c d
Video
RA cell SAN cell

Supplement: Supplementary file 2 — Supplementary Fig. 4 [file 12276_2023_988_MOESM2_ESM.pptx]
